# Supplementary material for: Bitis arietans Snake Venom Induces an Inflammatory Response Which Is Partially Dependent on Lipid Mediators
Source: Toxins (Basel). 2020 Sep 14;12(9):594. doi: 10.3390/toxins12090594 (PMC7551280; doi:10.3390/toxins12090594)
Supplement: Supplementary file 1 [file toxins-12-00594-s001.pdf]

Supplementary Materials: *Bitis arietans* Snake Venom Induces an Inflammatory Response Which is Partially Dependent on Lipid Mediators

Angela Alice Amadeu Megale, Fernanda Calheta Portaro and Wilmar Dias Da Silva

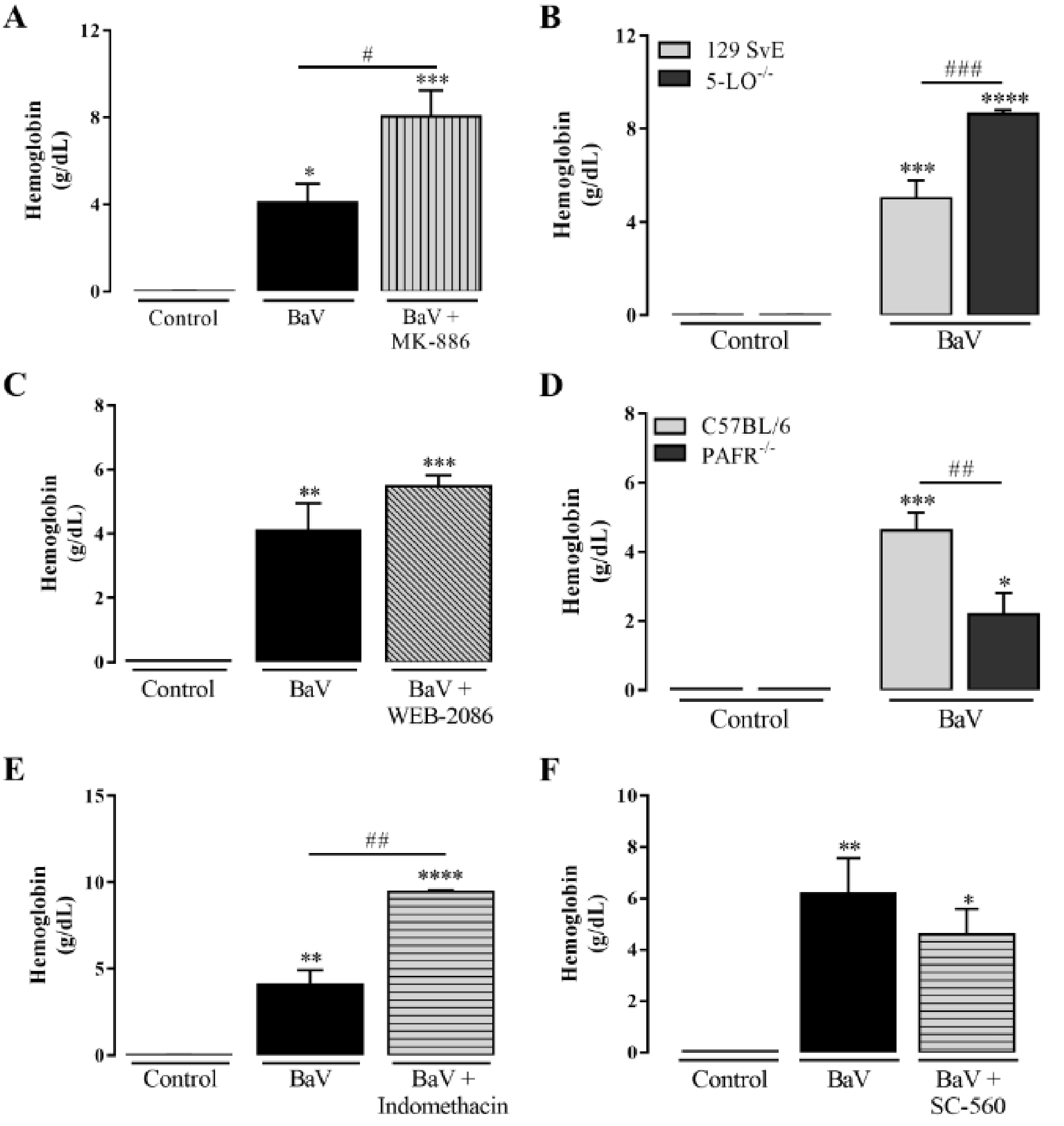

Figure S1. Local hemorrhage induced by BaV. Hemorrhage was evaluated in C57BL/6 mice pre-treated with anti-inflammatory drugs and in $5\text{-LO}^{-/-}$ and $\text{PAFR}^{-/-}$ genetically deficient mice. After 4 h of i.p. BaV inoculation (0.5 mg/kg), peritoneal exudate was harvested to evaluated the hemorrhage by hemoglobin quantification. The participation of leukotrienes was evaluated in: (A) mice pre-treated with MK-886 or (B)in mice genetically deficient of 5-LO ($5\text{-LO}^{-/-}$). The participation of PAFR was evaluated in: (**C**) mice pre-treated with WEB-2086 or (**D**) in mice genetically deficient of PAFR ($\text{PAFR}^{-/-}$). The participation of prostanoids was evaluated in mice pre-treated with: (**E**) Indomethacin; and (**F**) SC-560. (*) Differences between BaV and control; (#) differences between mice pre-treated or not with anti-inflammatory drugs and $5\text{-LO}^{-/-}$ and $\text{PAFR}^{-/-}$ mice compared to 129 SvE and C57BL/6 mice, respectively. Results were expressed as mean ± SD (4–5 mice) of three reproducible assays. (* or #) $p < 0.05$, (** or ##) $p < 0.01$, (*** or ###) $p < 0.001$ and (****) $p < 0.0001$.

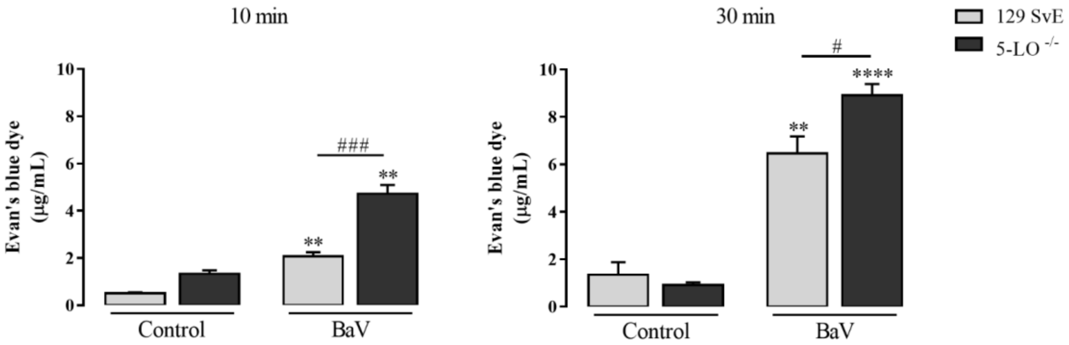

Figure S2. Vascular leakage in 129 SvE and 5-LO$^{-/-}$ mice. After 10 min and 30 min of i.p. BaV inoculation (0.5 mg/kg), peritoneal exudate was harvested to evaluate the increase in vascular permeability by Evan's Blue dye extravasation. (*) Differences between BaV and control; (#) differences between 129 SvE and 5-LO$^{-/-}$ mice. Results were expressed as mean ± SD (4–5 mice) of three reproducible assays. (#) $p < 0.05$, (**) $p < 0.01$, (###) $p < 0.001$ and (****) $p < 0.0001$.

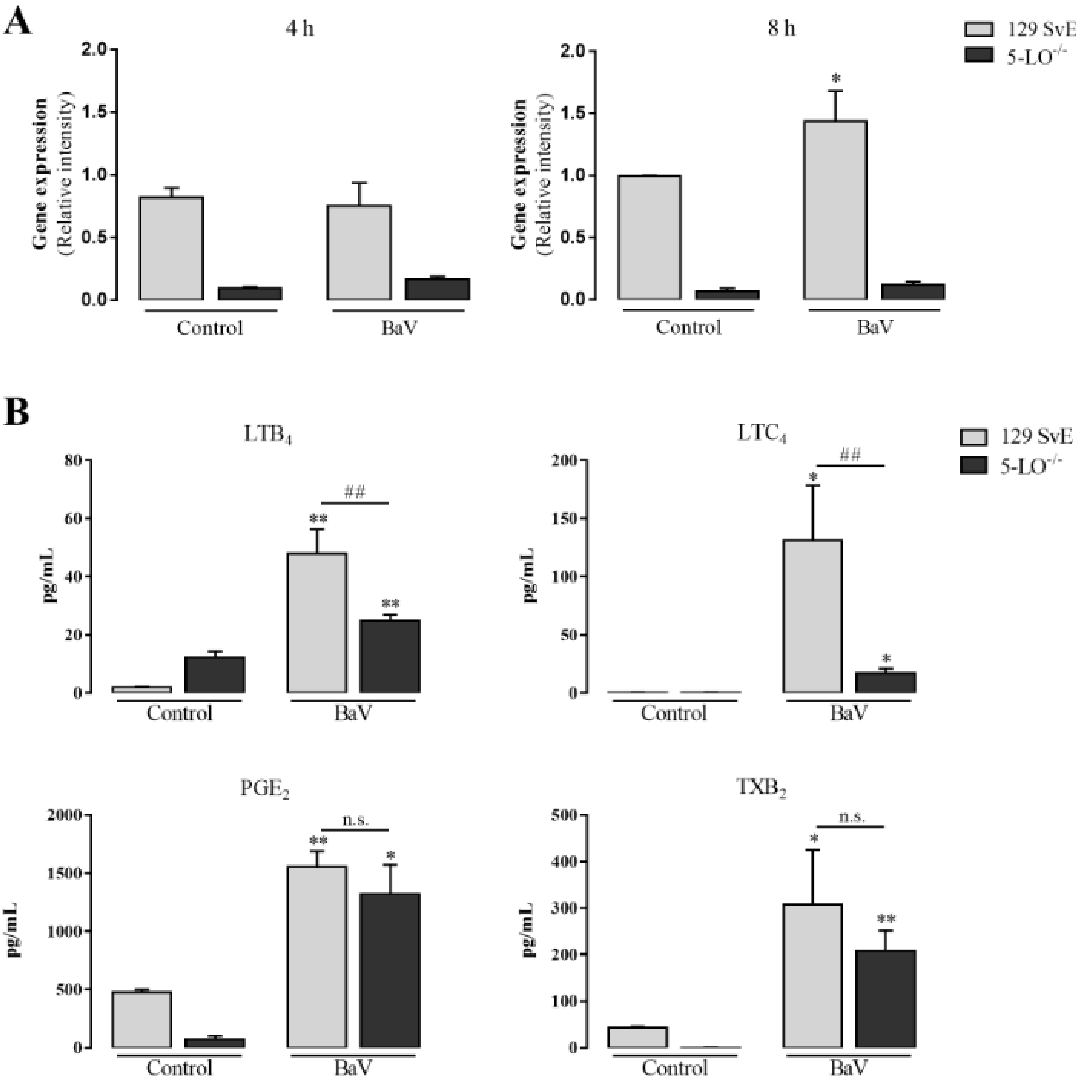

Figure S3. Expression of 5-LO enzyme and the production of lipid mediators in 5-LO$^{-/-}$ and 129 SvE mice. After selected periods of i.p BaV inoculation (0.5 mg/kg), peritoneal exudate was harvested to evaluate: (**A**) Gene expression of 5-LO after 4 h and 8 h; and (**B**) production of eicosanoids LTB_4, LTC_4, PGE_2 and TXB_2, after 30 min. (*) Differences between BaV and control; (#) differences between 129

SvE and 5-LO$^{-/-}$ mice. n.s.: not significant. Results were expressed as mean ± SD (4–5 mice) of three reproducible assays. (*) $p < 0.05$ and (** or ##) $p < 0.01$.

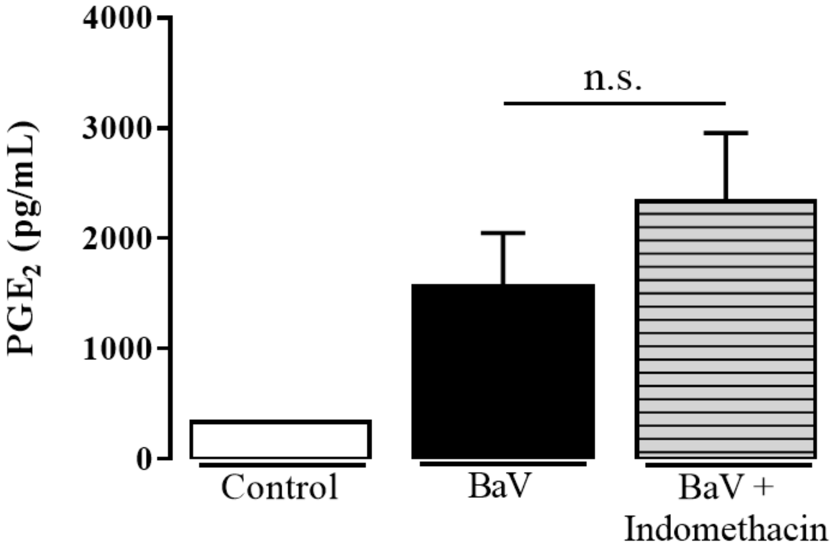

Figure S4. Production of PGE_2 after 4 h of BaV inoculation is not attenuate by indomethacin; 1 h before i.p. BaV inoculation (0.5 mg/kg), C57BL/6 mice were s.c. treated with indomethacin (10 mg/kg). After 4 h, peritoneal exudate was harvested to evaluate the production of PGE_2. n.s.: not significant. Results were expressed as mean ± SD (4–5 mice) of three reproducible assays.
